# Mitochondrial genome sequences from wild and cultivated barley (*Hordeum vulgare*)

**DOI:** 10.1186/s12864-016-3159-3

**Published:** 2016-10-24

**Authors:** Hiroshi Hisano, Mai Tsujimura, Hideya Yoshida, Toru Terachi, Kazuhiro Sato

**Affiliations:** 1 Institute of Plant Science and Resources, Okayama University, 2-20-1 Chuo, Kurashiki, Okayama 710-0046 Japan; 2 Plant Organelle Genomics Research Center and Faculty of Life Sciences, Kyoto Sangyo University, Motoyama, Kamigamo, Kita-ku, Kyoto 603-8555 Japan

**Keywords:** *Hordeum vulgare*, Mitochondrial genome, *De novo* assembly, Comparative genomics

## Abstract

**Background:**

Sequencing analysis of mitochondrial genomes is important for understanding the evolution and genome structures of various plant species. Barley is a self-pollinated diploid plant with seven chromosomes comprising a large haploid genome of 5.1 Gbp. Wild barley (*Hordeum vulgare* ssp. *spontaneum*) and cultivated barley (*H. vulgare* ssp. *vulgare*) have cross compatibility and closely related genomes, although a significant number of nucleotide polymorphisms have been reported between their genomes.

**Results:**

We determined the complete nucleotide sequences of the mitochondrial genomes of wild and cultivated barley. Two independent circular maps of the 525,599 bp barley mitochondrial genome were constructed by *de novo* assembly of high-throughput sequencing reads of barley lines H602 and Haruna Nijo, with only three SNPs detected between haplotypes. These mitochondrial genomes contained 33 protein-coding genes, three ribosomal RNAs, 16 transfer RNAs, 188 new ORFs, six major repeat sequences and several types of transposable elements. Of the barley mitochondrial genome-encoded proteins, NAD6, NAD9 and RPS4 had unique structures among grass species.

**Conclusions:**

The mitochondrial genome of barley was similar to those of other grass species in terms of gene content, but the configuration of the genes was highly differentiated from that of other grass species. Mitochondrial genome sequencing is essential for annotating the barley nuclear genome; our mitochondrial sequencing identified a significant number of fragmented mitochondrial sequences in the reported nuclear genome sequences. Little polymorphism was detected in the barley mitochondrial genome sequences, which should be explored further to elucidate the evolution of barley.

**Electronic supplementary material:**

The online version of this article (doi:10.1186/s12864-016-3159-3) contains supplementary material, which is available to authorized users.

## Background

Cultivated barley (*Hordeum vulgare* ssp. *vulgare*) was domesticated from its wild ancestral form (*H. vulgare* ssp. *spontaneum*) ca. 10,000 years ago [1, 2]. Cultivated and wild barley share closely related genomes that do not exhibit crossing or recombination barriers [3]. Barley is a self-pollinated diploid with seven pairs of chromosomes comprising a large haploid genome of 5.1 Gbp. The barley genome has been analyzed by BAC fingerprinting, BAC-end sequencing, whole-genome shotgun sequencing and transcriptome analysis [4]. The genome data also include haplotype sequence information for several cultivated and wild barley lines, which provides 15 million SNPs among haplotypes.

The possibility of multiple domestication events for barley has been discussed based on phylogenic analyses of cloned genes [2, 5, 6]. The wild ancestral form of barley is widely distributed in the Near East to Central Asia, with a center in the Fertile Crescent. During the evolution of wild barley, its mitochondrial genomes might have diversified. For example, Ahokas [7] detected cytoplasmic male sterility, one of the main traits derived from the mitochondrial genome, in a wild barley germplasm. This finding indicates that the organellar genomes have differentiated in wild barley during its long evolutionary process.

Although the gene contents of the mitochondrial genomes of flowering plants are nearly identical, gene order and genome configuration are highly variable even within a species due to genome rearrangement [8]. Tsunewaki et al. [9] explored the evolutionary dynamics of the wheat mitochondrial genome with respect to its structural differentiation during cereal evolution by comparing the nucleotide sequences of wheat genes to those of rice and maize.

The nuclear genome of barley is closely related to those of wheat and its relatives, i.e., members of the *Triticeae* tribe. The mitochondrial genome structure of common wheat (*Triticum aestivum*) cv. Chinese Spring is almost identical to that of the Chinese wheat cultivar Yumai, with differences of seven SNPs and 10 indels in non-coding regions [10, 11]. Noyszewski et al. [12] sequenced the *Triticeae* mitochondrial genomes of *Aegilops longissima*, *T. turgidum* and an alloplasmic line with the *Ae. longissima* cytoplasm that carries the *T. turgidum* nucleus, finding evidence suggesting that the alloplasmic condition accelerates evolution towards forming new mitochondrial genomes. However, to date, little information about the mitochondrial genome in barley has been published.

In a study investigating the chloroplast genome, which together with the mitochondrial genome composes the plasmon, Middleton et al. [13] found that the chloroplast sequences of cultivated and wild barley are closely related (sequence identity of 99.98 %). The divergence time of the two barley sequences was estimated to be 80,000 ± 20,000 years using semi-penalized likelihood. A comparison of the chloroplast genome sequences of cultivated barley and common wheat identified four deletions and five insertions greater than 50 bp in the chloroplast genome sequence of common wheat. This similarity in chloroplast sequence indicates that cultivated and wild barley are more closely related to each other than cultivated barley and cultivated wheat.

The haplotypes used in the current study were the Japanese malting barley cultivar ‘Haruna Nijo’ and the wild barley accession ‘H602’. Haruna Nijo has been extensively used as a crossing parent for developing high-quality malting barley cultivars in Japan. The origin of this cultivar can be traced to the haplotype of a malting cultivar introduced more than 100 years ago from Europe. Haruna Nijo has also been used to generate genomic resources, e.g., a BAC library [14], full-length cDNAs [15, 16], and whole genome shotgun sequence analysis [17]. Wild barley accession H602 has been used as a haplotype that is distantly related to Haruna Nijo and thus, a high-resolution transcript map of Haruna Nijo/H602 has been developed [18]. Both haplotypes may represent cultivated and wild barley gene pools, suggesting that they are appropriate materials for revealing the diversity of mitochondrial genomes in barley.

In this study, we determined the complete nucleotide sequences of the mitochondrial genomes of wild and cultivated barley and compared their structures. We then analyzed the gene contents and unique regions of each genome in detail at the sequence level. Since no mitochondrial genome sequence has been published for barley, we compared the resulting sequences to the published mitochondrial sequences of related species, i.e., wheat and its relatives, to clarify the evolutionary process of barley mitochondria.

## Methods

### Plant materials and mitochondrial DNA extraction

Mitochondrial DNA samples were extracted from etiolated seedlings barley cultivar ‘Haruna Nijo’ and wild barley accession ‘H602’ according to the method of Bonen and Gray [19], with minor modifications. Barley seeds were obtained from the National Bioresource Project of Barley, MEXT of Japan. Using discontinuous (1.15, 1.30 and 1.45 M) sucrose density gradient centrifugation, DNase-I treated mitochondria were collected from the interface between 1.30 and 1.45 M sucrose. The mitochondrial DNA samples were purified by EtBr/CsCl density-gradient ultracentrifugation. For diversity analysis of SNPs, seed samples from 246 barley cultivars and four wild accessions preserved at the Institute of Plant Science and Resources, Okayama University, Japan were used.

### Sequencing, assembly and completion

Both 454 sequencing with the GS-FLX Titanium platform (Roche Diagnostics K.K.), and Illumina sequencing with the MiSeq platform (Illumina, San Diego, CA, USA) were performed to generate longer reads by GS-FLX 454 system and correct read errors with high redundant shorter reads by MiSeq system. The libraries for 454 sequencing were prepared with GS Titanium Rapid Library Preparation Kit (Roche) and sequenced using GS FLX Titanium PicoTiterPlate Kit 70x75 (Roche) and GS FLX Titanium Sequencing Kit XL+ (Roche) following the manufacturer’s instructions. GS FLX Titanium Rapid Library MID Adapter Kit (Roche), GS FLX Titanium LV emPCR Kit (Lib-L) v2 (Roche) and GS FLX Titanium emPCR Breaking Kit LV/MV 12pc (Roche) were used for adapter ligation and emulsion PCR during library and sequencing preparation. The libraries for Illumina sequencing were prepared with Nextera XT DNA Sample Preparation Kit (Illumina) and Nextera XT index Kit (Illumina) and sequenced with MiSeq Reagent Kit v2 500 cycles (Illumina) following the manufacturer’s instructions.

The reads from 454 sequencing were assembled with Newbler 2.6 (Roche). The generated contigs were filtered by average depth (>15). BLASTN analysis was performed to exclude contamination of chloroplast genome sequences. Filtered contigs were temporally ordered by hooking shared terminator reads in each contig.

Modified touchdown PCR [20] was performed to connect the contigs and to validate the SNPs with the following program: initial denaturation at 98 °C for 2 min, 10 cycles of a melting step at 98 °C for 10 s, an annealing step at 65 °C decreased 1 °C/cycle for 15 s and an extension step at 72 °C for 1 min, followed by 20 cycles of denaturation at 95 °C for 10 s, annealing at 55 °C for 20 s and extension at 72 °C for 1 min, with a final extension at 72 °C for 10 min. The 10 μl reaction mixture for PCR included 1 ng mitochondrial DNA as template, 1× PrimeSTAR® Max DNA Polymerase (TAKARA, Japan) and 0.5 μM specific primers listed in Additional file 1: Tables S1a, b and Additional file 2: Tables S2. The PCR amplicons were checked by electrophoresis in 1.5 % agarose gels (Wako, Japan) in TBE running buffer. Direct sequencing of the PCR amplicons was performed on a 3130xl Genetic Analyzer (Applied Biosystems, Waltham, MA, USA).

To increase the accuracy of genome sequencing, Illumina-reads from H602 and Haruna Nijo were mapped and aligned onto the reference mitochondrial draft genome of H602.

### Genome annotation and comparative analysis

The genes encoding mitochondrial proteins and rRNAs were identified using BLASTN and MITOFY [21] based on the known annotation of the mitochondrial genes of wheat cv. Chinese Spring [10]. The tRNAscan-SE 1.21 server [22] was used to identify tRNAs. Repeat sequences (>100 bp and >80 % identity) were identified by Align Sequences Nucleotide BLAST using mitochondrial sequences from Haruna Nijo. Partial sequences sharing more than 10 % of nucleotides with the original genes were identified as pseudogenes. For transposable element analysis, CENSOR [23] was used with four sequence sources (database) including *Poaceae*, *Oryza*, *Panicoideae* and *Triticum*.

To identify novel ORFs, ‘getorf’ analysis in EMBOSS (http://emboss.sourceforge.net/apps/cvs/emboss/apps/getorf.html) was conducted with the following parameters: predicted protein more than 100 aa in length; methionine as the start codon; circular DNA as the query sequence. After getorf analysis, the ORFs overlapping with known genes were eliminated.

### Alignments and phylogenetic analysis

Phylogenetic analysis was carried out using concatenated nucleotide sequences of the 24 protein-coding genes (all electron transport chain genes and *ccmB*, *ccmC*, *ccmFC*, *ccmFN*, *matR* and *mttB*) extracted from the complete mitochondrial genome sequences deposited in the public databases. All positions containing gaps and missing data were eliminated, yielding a total of 23,875 positions in the final dataset. Alignments and phylogenetic tree construction were performed by the maximum likelihood method with the MEGA5 program [24].

### SNP analysis

A modified touchdown PCR procedure, as described previously, was performed for SNP analysis with specific primer pairs (Additional file 2: Table S2) using 50 ng genomic DNA from cultivated and wild accessions of barley. PCR amplicons were sequenced with a 3130xl Genetic Analyzer.

## Results

### Sequencing and assembly of the mitochondrial genome in barley

The mitochondrial genomes of wild and cultivated barley were analyzed by 454 sequencing (Roche Diagnostics K.K.). A total of 954,203 and 256,123 reads corresponding to 280 Mbp (average read length 586 bp) and 132 Mbp (514 bp) were generated from H602 and Haruna Nijo, respectively. These reads were independently *de novo* assembled using Newbler 2.6 (Roche) with average contig sizes of 993 and 1624 bp, N50 contig sizes of 915 and 1789 bp and largest contig sizes of 76,676 and 56,491 bp for H602 and Haruna Nijo respectively. After trimming of duplicated sequences, these contigs were aligned in 18 contigs (525,551 bp) for H602 and 27 contigs (525,569 bp) for Haruna Nijo (Additional file 3: Tables S3a and S3b). The contig sequences were aligned, followed by sequencing of the PCR products with a 3130xl Genome Analyzer (see Additional file 1: Tables S1a and S1b for primer information). Draft master circular maps (master circles) of the mitochondrial genome were generated for both H602 and Haruna Nijo.

The Illumina MiSeq reads were mapped onto the master circles and re-aligned to develop high quality mitochondrial sequences. From the mitochondrial sequencing libraries of H602 and Haruna Nijo, 2,241,878 and 6,973,796 paired reads were generated, respectively. Reads of H602 were trimmed with CLC genomic workbench 8.0 (Filgen Inc.), and the resulting pairs of 596,175 reads were mapped onto the draft master circle of H602. A 525,599 bp circular molecule with a G/C content of 44.2 % (accession no. AP017300) was developed for H602. Using the same methods, another 525,599 bp circular molecule (accession no. AP017301) was developed for Haruna Nijo with pairs of 2,119,807 trimmed reads.

The alignment of two mitochondrial circular molecules identified three SNPs between H602 and Haruna Nijo at the positions of nt 510 (A/T, SNP1), 193,306 (G/T, SNP2) and 292,434 (A/C, SNP3). SNP1 was located in a genic region (*rps3a_p*), and SNP2 and SNP3 were located in intergenic regions. These SNPs were validated by Sanger sequencing of PCR products, including the alleles amplified by specific primers (Additional file 2: Table S2).

### Annotation of the mitochondrial genome

**Fig. 1:**
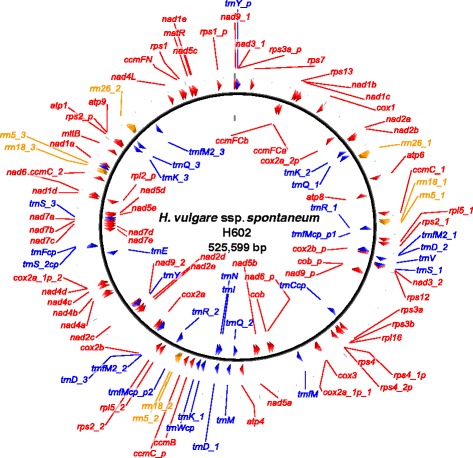
Positions of genes on the barley mitochondrial genome map (*Hordeum vulgare* ssp. *spontaneum* acc. H602). Genes encoding proteins (*red*), rRNA (*orange*) and tRNA (*blue*) are shown outside (forward direction) and inside (reverse complementary direction) the circle. *Arrowhead* indicates gene or exon, *p* indicates pseudogene, *a*, *b*, *c*, *d* and *e* indicate cis- and trans-spliced genic exon and underscores followed by numbers indicate gene copies

The master circles were annotated by BLAST analysis against the mitochondrial genome annotation databases MITOFY [21] and tRNAscan-SE 1.21 [22], and 33 protein-coding genes, three ribosomal RNAs and 16 transfer RNAs were identified (Fig. 1 and Additional file 4: Table S4). Of the 33 protein-coding genes, 18 encoded members of the electron transport chain and ATP synthase, including the following: nine subunits of complex I including *nad1*, *nad2*, *nad3*, *nad4*, *nad4L*, *nad5*, *nad6*, *nad7* and *nad9*; an apocytochrome b subunit (*cob*) of complex III; three subunits of complex IV including *cox1*, *cox2* and *cox3*; and five subunits of complex V including *atp1*, *atp4*, *atp6*, *atp8* and *atp9*. Four additional protein-coding genes were involved in cytochrome c biogenesis, including *ccmB*, *ccmC*, *ccmFC* and *ccmFN*. There were 10 genes for ribosomal proteins, namely *rps1*, *rps2*, *rps3*, *rps4*, *rps7*, *rps12*, *rps13*, *rpl5*, *rpl16* and pseudogene *rpl2p*, as well as two other protein-coding genes, *matR* and *mttB*. Among the protein-coding genes, there were two copies of *nad3*, *nad9*, *ccmC*, *rps2* and *rpl5*. Among rRNA and tRNA genes, there were three copies of *rrm5*, *rrn18*, *trnD*, *trnfM2*, *trnK* and *trnQ* and two copies of *rrn26* and *trnR*. Of the two copies of *cox2b*, the one nearest to *cox2a* was regarded as active *cox2b*, whereas the other, which is located far from *cox2a*, was regarded as a pseudogene due to the general rule of cis-splicing between *cox2a* and *cox2b*.

### Prediction of new genes

We used the ‘getorf’ command in EMBOS to identify unique genes in the barley mitochondrial genome, leading to the prediction of 188 new ORFs (>100 amino acids). After excluding any partially overlapped ORFs with known genes in the same translational frame, 142 ORFs were identified as novel genes (Additional file 5: Table S5). The total length of these novel ORFs was 66,180 bp, which occupied 12.6 % of the barley mitochondrial genome.

### Identification of transposable fragments

CENSOR [23] was used to identify integrated transposable elements showing similarities to the sequences in the *Poaceae* (grass family), *Oryza* (rice), *Panicoideae* (maize, sugarcane, sorghum and millet) and *Triticum* (wheat, barley and rye) databases. A total of 88 (15,202 bp total) DNA transposons, LTR retrotransposons and non-LTR retrotransposons were found in *Poaceae*, 59 (9586 bp) were found in *Oryza* and 73 (2682 bp) were found in *Panicoideae* (Table 1). By contrast, only 16 LTR retrotransposon and two non-LTR retrotransposons (cumulatively 2246 bp) were found in *Triticum*.

**Table 1:** Transposable elements in the barley mitochondrial genome

	*Poaceae* ^a^		*Oryza* ^a^		*Panicoideae* ^a^		*Triticum* ^a^	
Repeat class	No. of fragments	Length	No. of fragments	Length	No. of fragments	Length	No. of fragments	Length
**DNA transposon**	**13**	**837**	**9**	**522**	**8**	**406**	-	-
EnSpm	1	58	1	97	2	93	-	-
Harbinger	1	38	1	38	-	-	-	-
Helitron	5	428	2	157	-	-	-	-
MuDR	5	277	-	-	5	277	-	-
hAT	-	-	4	194	-	-	-	-
Other	1	36	1	36	1	36	-	-
**LTR retrotransposon**	**57**	**10,210**	**41**	**572**	**49**	**8591**	**16**	**2043**
Copia	19	3574	10	1947	14	2306	7	890
Gypsy	38	6636	31	6139	35	6285	9	1153
**Non-LTR**	**18**	**4155**	**9**	**978**	**16**	**3685**	**2**	**103**
L1	17	3911	6	572	16	3685	1	41
Other	1	244	3	406	-	-	1	62
**Total**	**88**	**15,202**	**59**	**9586**	**73**	**12,682**	**18**	**2146**

^a^Sequence sources (database) for transposable element analysis in CENSOR

### Repeat sequences and chloroplast-derived region in the mitochondrial genome

**Fig. 2:**
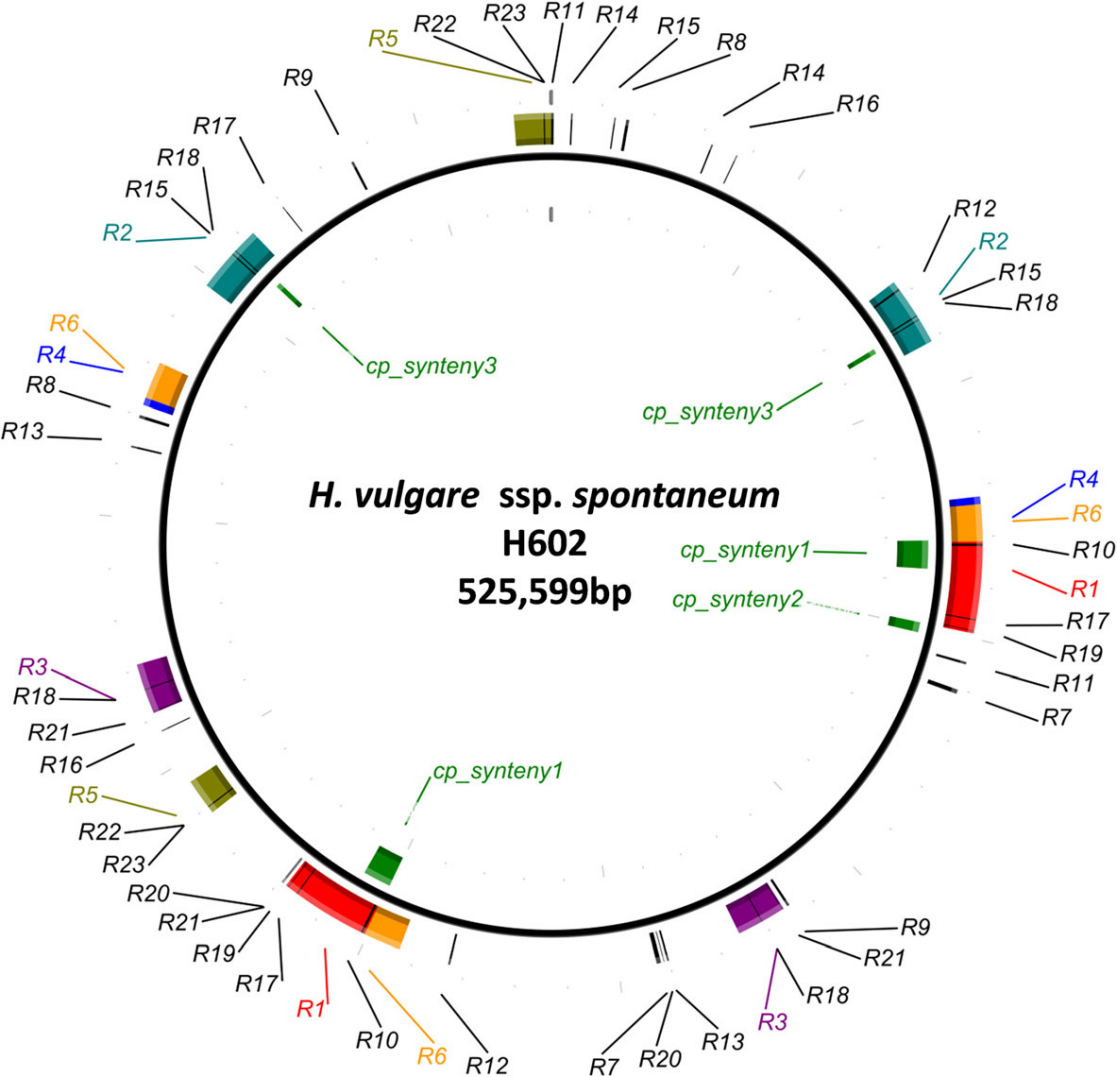
Repeat sequences and integrated chloroplast genome in the map of the barley mitochondrial genome of accession H602. Repeat sequences in different colors are mapped onto the barley mitochondrial genome (*outside*). Detailed information about these repeats is shown in Additional file 6: Table S6. The predicted sequences integrated from the chloroplast genome are shown inside the circle

Six major repeat sequences (>1000 bp, identity 100 %) and 17 repeat sequences (>100 bp, identity >99 %) were found in the barley mitochondrial genome (Fig. 2 and Additional file 6: Table S6). There were four repeats in No. 18 and three in Nos. 6, 12, 15, 17 and 21. The largest repeat was 25,303 bp, corresponding to the contig_H602_0022-0039-0169-0014 (Additional file 3: Tables S3a and S3b).

According to previous reports [25, 26], several tRNAs that function in the mitochondria originated in the chloroplast. In the barley mitochondrial genome, *trnC*, *trnF*, *trnS2* and *trnW* could represent tRNA genes of chloroplast origin (Additional file 4: Table S4). We analyzed the sequence similarity between the mitochondrial genome of H602 and the chloroplast genomes [13] by BLASTN analysis of barley cvs. Barke (KC912687) and Morex (EF115541) and found three homologous regions (>1 kbp) (Additional file 7: Table S7). Homologous region 1, harboring *trnfM*, *trnR*, *rps14* and *psaA*, contained 6307 bp in the R1 repeat region (Additional file 6: Table S6), suggesting that the mitochondrial genome contained two copies of homologous region 1. Among these genes, *trnfM* and *trnR* were identified using the mitochondrial genome annotation database, whereas *rps14* and *psaA* were not identified. Homologous region 2 was 2265 bp in length and harbored *trnV*. Homologous region 3 contained 1110 bp of sequences specific to the chloroplast sequence of Barke. Although homologous regions 1 and 2 were found in the chloroplast genomes of several monocot species such as rice and maize, region 3 was only found in Barke. Fifteen other homologous regions, ranging from 29 to 558 bp in length, were also found by BLASTN analysis (Additional file 7: Table S7).

### Transfer of mitochondrial DNA into the barley nuclear genome

We analyzed nucleotide sequence similarities between the mitochondrial genome of H602 and the published barley genome sequences (Hordeum_vulgare.ASM32608v1.31.dna_sm.genome.fa.gz), comprising pseudomolecules of seven chromosomes and un-anchored scaffolds and contigs published in Ensembl Plants *Hordeum vulgare* (http://plants.ensembl.org/Hordeum_vulgare/Info/Index), using BLASTN analysis (Additional file 8: Table S8). Using the threshold E-value = 0, aligned sequence length > 400 bp, we detected 621 barley genome sequences showing similarities to the barley mitochondrial genome sequence. Of these, many of the longest un-anchored genome sequences appeared to be mitochondrial sequences, since the lengths of un-anchored sequences and aligned sequences were quite similar. For example, the sequence of morex_contig_70567, which was 23,224 bp in length, aligned completely with the mitochondrial sequence without mismatches or gaps. Of the genome sequences within the threshold, seven were derived from the chloroplast genome and 93 were un-anchored contigs that were potentially derived from the mitochondrial genome in the published barley genome sequence obtained by whole-genome shotgun sequencing. Apart from the misidentified mitochondrial sequence, nuclear sequences of mitochondrial origin (Nuclear mitochondrial DNA = NUMTs) were observed. Of the 561 possible NUMTs anchored to pseudomolecules, 11,579 bp of sequence on chromosome 6H was the longest that aligned with the mitochondrial genome. The total amounts of aligned sequences varied among chromosomes, ranging from 16,427 bp (5H) to 150,387 bp (3H), indicating that the mitochondrial sequences were not evenly transferred to the nuclear genome [27].

### SNP analysis of barley germplasm

Barley germplasm comprising 246 cultivars from 40 regions (countries) and four wild accessions were genotyped at three SNPs that were identified between the H602 and Haruna Nijo mitochondrial genomes. Of the 188 cultivars showing a nucleotide call on SNP1 (H602/Haruna Nijo: A/T), 13 and 175 contained an A and T, respectively (Additional file 9: Table S9). Of the four wild barley accessions, two contained A and two contained T. For SNP2 (T/G), all of the cultivars and three wild barley accessions contained G, but one wild barley, H627, contained T. For SNP3 (C/A), 184 of 223 cultivars and one wild barley (H685) contained A (Additional file 9: Table S9). Most Ethiopian cultivars contained C in SNP3, although other regional differences in the allele distribution were not obvious.

### Comparative genomics of barley mitochondria with those of wheat and other monocot species

**Fig. 3:**
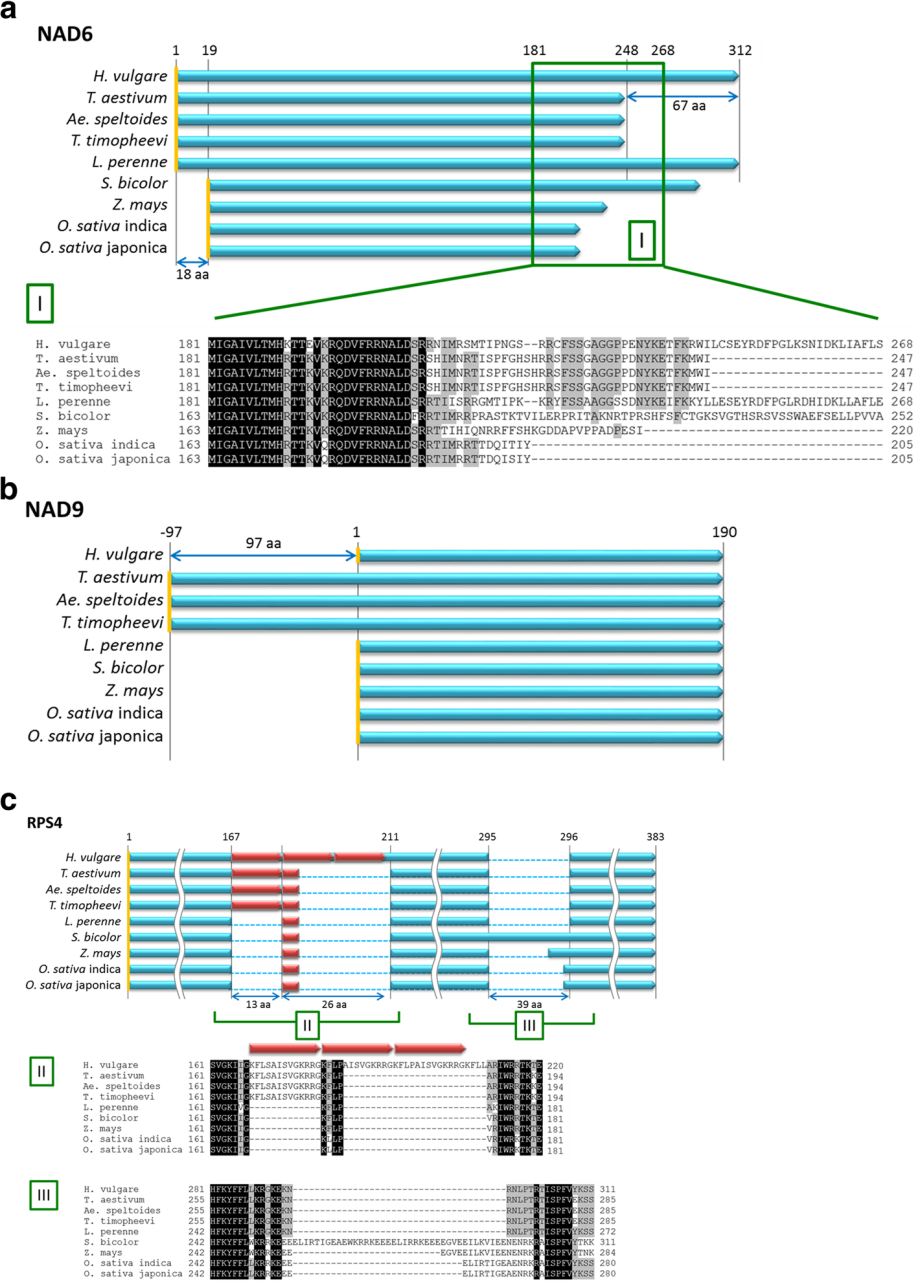
Structures of the encoded protein sequences in various grass species. The structures of **a** NAD6, **b** NAD9 and **c** RPS4 in the barley mitochondrial genome were compared with those of seven other grass species and subspecies (*T. timopheevii*, *Ae. speltoides*, *L. perenne*, *S. bicolor*, *Z. mays*, *O. sativa* ssp*. japonica* and *O. sativa* ssp. *indica*). *Light blue* bars show the primary structures of proteins, and *orange lines* indicate the start codon. The numbers above the *light blue bars* indicate the position from the start codons of barley proteins. **a** *Green box I* indicates the non-conserved amino acid sequences in NAD6 among grass species. **b** NAD9 of *T. aestivum*, *Ae. speltoides* and *T. timopheevii* have an additional 97 aa on their N-terminal side compared with those of other grass species including barley. **c** *Red bars* indicate 13 aa repeats. *Green boxes II and III* indicate species-specific amino acid sequences in RPS4

We compared the genes/proteins in the barley mitochondrial genome with those in other grass species including common wheat (*T. aestivum*) and wheat ancestors (*T. timopheevii* and *Ae. speltoides*), perennial ryegrass (*L. perenne*), sorghum (*S. bicolor*), maize (*Z. mays*) and rice (*O. sativa*). Barley *nad6* encoded NAD6, a 312 aa polypeptide (Table 2,  Fig. 3 and Additional file 10: Figure S1). The sequence comprising aa 1 to 215 in NAD6 was conserved among grass species, whereas the C-terminus is highly variable. Barley *nad9* was conserved among grass species except wheat, which contained a longer *nad9* due to frame-shifting caused by a 4 nt insertion/deletion (Table 2, Fig. 3b and Additional file 10: Figure S1). The *rps4* gene had 39-nt imperfect repeats corresponding to 13 aa (Fig. 3c and Additional file 10: Figure S1). Barley *rps4* had three repeats, whereas wheat has one and rice, maize and perennial ryegrass each have partial one (Fig. 3c). The genes *cox1*, *atp6*, *rps2*, *rpl5* and *rrn18* also varied between barley and wheat (Table 2).

**Table 2:** Differences in mitochondrial genes encoding proteins between barley and wheat

Gene in barley	Differences from wheat
*nad6*	1. Low homology at 3′ side (from 646 to 738 nt).
	2. Additional 201 nt on 3′ side (67 amino acid on the C-terminus) because of stop codon substituted to leucine caused by A to T substitution at 737 nt.
*nad9*	Deficient of 97 aa on 5′ side, however identical to ones in rice, bamboo, maize, sorghum and ryegrass.
*cox1*	Two additional C-terminal aa.
	Wheat *cox1* is the same as that of rice and bamboo.
*atp6*	Low homology at 5′ side^a^.
*rps2*	Low homology at 3′ side^b^.
*rps4*	Insertion of 78 nt (26 aa) caused by multiple repeat sequences.
	Barley has three of 39 nt repeat sequence, whereas wheat has one of this sequence.
*rpl5*	Difference of 10 aa on C-terminus.
	Wheat *rpl5* is same as that of rice, bamboo and ryegrass.
*rrn18*	Insertions of 18 nt and 4 nt.

^a^Bonen and Bird [36] indicated that the N-terminus of ATP6 in the mitochondrial genome had interspecies variation caused by processing

^b^Kubo et al. [37] reported RPS2 in barley (BAD61010) and interspecies variation in *Triticeae* at its C-terminus

**Fig. 4:**
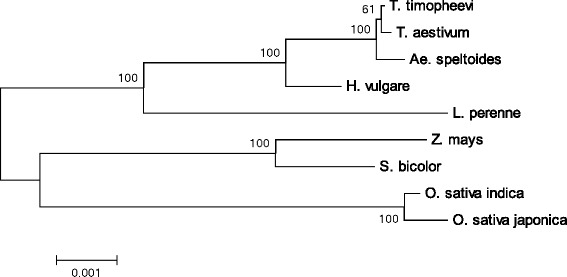
Phylogenetic tree constructed using the DNA sequences of 24 protein-coding genes comprising a total of 23,875 bp in the mitochondrial genomes of eight grass species and subspecies by the maximum likelihood method. The protein-coding genes include all electron transport chain genes, *ccmB*, *ccmC*, *ccmFN*, *matR* and *mttB*. The sequences of these genes can be found under the following accession numbers: AP013106 (*T. timopheevii*), AP013107 (*Ae. speltoides*), JX999996 (*L. perenne*), DQ984518 (*S. bicolor*), AY506529 (*Z. mays*), BA000029 (*O. sativa* ssp. *japonica*) and DQ167399 (*O. sativa* ssp. *indica*). The percentage of trees in which the associated taxa clustered together in 1000 replicates of the bootstrap test is shown next to the branches. *Scale bar* indicates the number of base substitutions per site

We analyzed the evolutionary relationships among grass species by examining the DNA sequences of protein-coding genes in the mitochondrial genome. A phylogenetic tree constructed in MEGA5 is shown in Fig. 4. Barley belonged to the *Triticeae* clade, which included *T. aestivum*, *T. timopheevii* and *Ae. speltoides. L. perenne* was placed closer to barley than to *S. bicolor*, *Z. mays* and *O. sativa*, which belonged to another clade.

## Discussion

### Features of the barley mitochondrial genome

In this study, we determined the complete nucleotide sequences of mitochondrial genomes from the wild barley accession H602 and the Japanese malting cultivar Haruna Nijo. These two haplotypes differ in origin and have sufficient polymorphisms in the nuclear DNA sequences for a proper comparison [28]. However, we detected a high level of sequence identity (only three SNPs in 525,599 bp) between the two mitochondrial genomes; the mitochondrial genomes of these wild and cultivated barley lines were almost identical in terms of both nucleotide sequence and genome structure.

Our annotation indicated that the barley mitochondrial genome contains a total of 96 genes (51,476 bp, excluding pseudogenes; Additional file 4: Table S4). These genes share similar components with genes in the wheat mitochondrial genome [10], as described in the Results and Additional file 4: Table S4. ORF prediction also suggested that the genome contains 142 ORFs (66,180 bp in total) comprising 16 % of the total genome (Additional file 5: Table S5). Six types of repeated sequences (R1 to R6) larger than 1 kbp were found in the barley mitochondrial genome (Additional file 6: Table S6). The repeated sequences were 100 % identical to each other and are located in direct orientation. There were two copies of R1 to R5 and three copies of R6 (Additional file 6: Table S6). Using CENSOR analysis, we identified a series of transposon types (Table 1). The total sequence lengths of *Copia*, *Gypsy* and non-LTR retrotransposons in the mitochondrial genome were 3574, 6636 and 4155 bp, respectively. In the nuclear genome of barley (63 Mbp BAC end sequences, [4]), ca. 81.80 % is occupied by mobile elements, with a retroelement/DNA transposon ratio of 12.16 %. Of these, the ratios of *Copia*, *Gypsy* and non-LTR retrotransposons are 13.66, 20.84 and 0.31 %, respectively. We found the retroelement/DNA transposon ratio in the mitochondrial genome to be 17.16 %, which is much higher than that of the nuclear genome. The barley mitochondrial genome appears to have more *Gypsy* elements and many more non-LTR retrotransposons than the nuclear genome.

The barley mitochondrial genome contained three chloroplast-homologous sequences (6307, 2265 and 1110 bp) larger than 1 kbp (Additional file 7: Table S7). Of these, two larger sequences were homologous to the chloroplast sequences of five barley cultivars [13] and even to those in wheat and rye. This result indicates that these homologous sequences were transferred from the chloroplast genome to the mitochondrial genome before the divergence of barley from a wheat/rye lineage. Another region (1110 bp) showed sequence similarity only to the chloroplast genome of barley cv. Barke. This 1110 bp sequence could be cultivar specific, and its transfer from the mitochondrial genome to the chloroplast genome might have occurred quite recently only in this cultivar. However, gene transfer between genomes is usually restricted to transfer from the chloroplast and mitochondria to the nucleus, known as nupt and numt, respectively, and from the chloroplast to mitochondria. It is highly improbable that we encountered a rare transfer event from the mitochondria to chloroplasts in this cultivar. It is likely, therefore, that this 1110 bp mitochondrial sequence was mis-assembled with the chloroplast genome of cv. Barke. The chloroplast-homologous sequence of 6307 bp in the barley mitochondrial genome was longer than the corresponding sequence in the wheat mitochondrial genome, and annotation information (Additional file 4: Table S4) revealed that the tRNAs *trnR* and *trnfM* were included in this fragment. In addition, the 2265 bp sequence contained *trnV*. Perhaps these chloroplast-derived tRNAs function in the barley mitochondria, although experimental evidence is needed to support this notion.

### Diversity of barley mitochondrial sequences

In the present study, only a few nucleotide polymorphisms were found between wild barley and cultivated barley mitochondrial haplotypes. Barley cultivation has a history of ca. 10,000 years after domesticated from its wild progenitor. According to the estimation of domestication mutation events on the *Btr1* (*brittle rachis 1*) and *Btr2* loci, which are responsible for the reproductive barrier via seed dispersal [2], there are two main sources of cultivated barley ancestors. It is surprising that the organellar genome of the wild haplotype used in this study was quite similar to that of the cultivated barley Haruna Nijo.

Of the SNPs detected in four wild and 246 cultivated barleys, only one wild barley showed an identical haplotype to H602 that was not found in the cultivated barleys examined (Additional file 9: Table S9). Interestingly, the haplotype in Haruna Nijo was also detected in wild barley accession H685. Among the three SNPs, the H602 allele of SNP3 was abundant in North African and Ethiopian landraces, indicating that SNP3 can be traced to the germplasm distributed in North Africa, with an origin related to wild barley accession H695. The haplotype with the H602 alleles in SNP1 and SNP3 was also present in both wild and cultivated barley. Since there is no crossing barrier between the wild ancestral form and cultivated barley, it is possible that the mitochondrial genome of H602 resulted from hybridization between wild and cultivated barley. More extensive sequencing of the mitochondrial genome is needed to elucidate mitochondrial genome evolution in wild and cultivated barley.

### Genome integration

Wicker et al. [29] reported that the mitochondrial genome was included in the nuclear genome, as revealed by low-redundancy shotgun genome sequencing. According to BLASTN sequence similarity analysis (threshold: E = 0, alignment length > 400 bp) in the present study, many of the published genome sequences shared similarity with the mitochondrial genome (Additional file 8: Table S8). The length of total integration of the mitochondrial genome into the chromosome-anchored nuclear genome sequence was 595,866 bp, which is estimated to represent 0.01 % of the total nuclear genome size in barley (5.1 Gbp). However, these mitochondrial sequences were not distributed evenly on the chromosomes, indicating that there are hot spots of chromosomal regions where mitochondrial sequences had transferred (Additional file 8: Table S8). The mitochondrial genome shared similarity with many of the long un-anchored sequences, which could represent sequences corresponding to the mitochondrial genome itself generated from whole-genome shotgun sequencing. The results also suggested that the plastid genome shares 18,215 bp of sequence with the mitochondrial genome, which had three duplicated regions corresponding to 7.54 % (10,240 bp) of the total plastid genome (135,802 bp). Thus, the current complete barley mitochondrial genome sequence represents an important tool for annotating the nuclear genome sequence, as well as the mitochondrial sequence itself spuriously included in the previously reported nuclear genome sequence.

### Comparative genomics with other crops

The size of the barley mitochondrial genome was 16 and 7 % larger than that of wheat (452,528 bp for cv. Chinese Spring) and rice (490,520 bp for cv. Nipponbare), respectively, but 30 % smaller than that of perennial ryegrass (678,580 bp). The barley mitochondrial genome was comparable in size to those of maize (535,825 and 557,162 bp for CMS-T and CMS-S type, respectively, and 569,630 bp for NB type from the inbred line B37N). Multiple genome alignment analysis using progressiveMauve [30] revealed the presence of more than 70 short syntenic regions between barley and wheat (data not shown). This complex pattern of fragmented synteny prevented us from reconstructing the evolutionary process by which extensive rearrangements occurred between the two mitochondrial genomes.

By contrast, a high proportion of genes in the mitochondrial genome of barley were almost the same as those of other grass species. In particular, the nucleotide sequences of protein-coding genes of the electron transport chain were highly conserved among grasses, although the presence of a few variations allowed us to construct a phylogenetic tree showing clear differentiation between *Triticeae* and other grass species (Table 2 and Fig. 3). Figure 4 shows a phylogenetic tree reconstructed from the nucleotide sequences of protein coding genes in the electron transport chain and those that function in cytochrome c biogenesis, as well as *matR* and *mttB*. The tree indicates that barley is relatively closely related to wheat, as expected. The analysis using the program MITOFY also shows the sequences of individual genes of barely are closely related to those of corresponding genes of wheat. Therefore, there is no indication that any genes of barely have derived from other unrelated plant species by horizontal gene transfer.

Based on the analysis of the sequences of nuclear genes encoding multi-domain plastid acetyl-CoA carboxylase (ACCase) and plastid 3-phosphoglycerate kinase (PGK), it was estimated that *Hordeum* diverged from the *Triticum*/*Aegilops* lineage ca. 11 million years ago (MYA) [31]. On the other hand, comparative chloroplast genome analysis led to the estimate that barley diverged from rye and wheat approximately 8–9 MYA [13]. Since barley shares a homoeologous chromosome system with wheat, only translocations differentiate the barley and wheat genomes.

Although the gene contents in the barley mitochondrial genome were quite similar to those of wheat [10], three genes (*nad6*, *nad9* and *rps4*) differed considerably between barley and wheat in terms of structure (Table 2, Fig. 3 and Additional file 10: Figure S1). Compared to wheat *nad6*, barley *nad6* contained a 198 nt extension in the 3′ coding region. This extension may produce a NAD6 polypeptide that is 66 aa longer in barley than in wheat. Although the length of NAD6 deduced from the genome sequence of perennial ryegrass was nearly identical to that of barley, significant variations in both length and sequence were observed in the C-terminal half of NAD6 among grass species (Fig. 3a). NAD6 is a hydrophobic membrane protein and a component of the NADH dehydrogenase complex (complex I). Complex I consists of approximately 50 proteins, seven subunits of which are encoded by the mitochondrial genome [32]; it is difficult to imagine that NAD6 variants are properly integrated into such a sophisticated molecular machinery. To investigate the possibility that all grasses actually contain a short conserved NAD6, we looked for a potential stop codon before an in-frame stop codon in each *nad6* gene that might have been created by RNA editing. Sequence comparison, however, showed no clear indication that a new stop codon was created and that premature termination helped increase the similarity among NAD6 polypeptides. It should be noted, however, that *nad6* could be expressed from mRNAs lacking a stop codon in *Arabidopsis* [33]. In *Arabidopsis* (and cauliflower), *nad6* mRNA was processed upstream of the in-frame stop codons, and the resulting mRNAs lacking stop codons were translatable [33]. This observation suggests that mRNA processing occurs at some points before an in-frame stop codon and that processing cancels apparent length variations in *nad6* genes among grass species. If this occurs, highly similar NAD6 polypeptides will be translated and integrated into complex I of each grass species. Determining the amino acid sequence constituting the C-termini of NAD6 from barley and other grasses will help test this notion.

The *nad9* gene from barley shared identical sequences with those of most grass species. An exception was wheat *nad9*, which is 291 nt longer than *nad9* genes from barley and other species. Two possible initiation codons (ATG) are present in the 5′ region of wheat *nad9*, and if the second ATG is used for translation, as suggested by Lamattina et al. [34], a similar polypeptide to that of barley will be produced in wheat.

The *rps4* gene contains variable numbers of a 39 nt repeat, depending on the species; this repeat is categorized as a minisatellite. Some plant mtDNAs contain minisatellites, most of which are located in intergenic regions, although a few occur in gene-coding and intronic regions [35]. The size of the abovementioned minisatellite (39 nt) is a multiple of three, which may explain why this minisatellite has survived in the coding region of *rps4* even after the divergence of the barley/wheat lineages. Since the variability of minisatellites is generally high, such minisatellites can be used to find polymorphisms among the mitochondrial genomes of wild and cultivated barley.

## Conclusions

The mitochondrial genome of barley was similar to those of other grass species in terms of gene content, but the configuration of the genes was quite different from that of wheat, which was used as a reference. Our data show that mitochondrial genome information is essential for correct annotation of the barley nuclear genome. Analysis of our newly generated mitochondrial sequence identified a significant number of fragmented mitochondrial sequences in the nuclear genome, plastid genomes and (importantly) un-anchored genomic sequences. The small polymorphisms in the mitochondrial genome sequences between the wild and cultivated barley lines examined in this study should be further explored to help confirm that the diversity of barley mitochondrial genome sequences is indeed small. Alternatively, we may have chosen samples with similar gene pools in terms of mitochondrial variation.

## Additional files

Additional file 1: Table S1. a Primers used for gap filling in the H602 mitochondrial genome, b Primers used for gap filling in the Haruna Nijo mitochondrial genome. (XLSX 14 kb)
Additional file 2: Table S2. Primer pairs used for SNP analysis. (XLSX 9 kb)
Additional file 3: Table S3. a Contigs of the mitochondrial genome of H602 generated by 454 sequencing, b Contigs of the mitochondrial genome of Haruna Nijo generated by 454 sequencing. (XLSX 13 kb)
Additional file 4: Table S4. Genes identified in the mitochondrial genome of barley. (XLSX 18 kb)
Additional file 5: Table S5. Novel ORFs in the barley mitochondrial genome. (XLSX 15 kb)
Additional file 6: Table S6. Repeated sequences in the mitochondrial genome of H602. (XLSX 15 kb)
Additional file 7: Table S7. Syntenic region between the mitochondrial genome and chloroplast genome in barley. (XLSX 12 kb)
Additional file 8: Table S8. BLASTN analysis between the mitochondrial sequences of H602 and pseudomolecules of seven chromosomes and un-anchored scaffolds and contigs published in Ensembl Plants *Hordeum vulgare* (Hordeum_vulgare.ASM32608v1.31.dna_sm.genome.fa.gz). (XLSX 59 kb)
Additional file 9: Table S9. SNP genotypes of 5 wild and 247 cultivated barleys. (XLSX 22 kb)
Additional file 10: Figure S1. Alignment of protein sequences encoded in the mitochondrial genomes of grass species. (DOC 740 kb)

## Abbreviations

BAC: Bacterial artificial chromosome
ORF: Open reading frame
PCR: Polymerase chain reaction
SNP: Single nucleotide polymorphism
