# Supplementary material for: Mitochondrial genome sequences from wild and cultivated barley (Hordeum vulgare)
Source: BMC Genomics. 2016 Oct 24;17:824. doi: 10.1186/s12864-016-3159-3 (PMC5078923; doi:10.1186/s12864-016-3159-3)

**Supplemental figure legend**

Figure S1. Alignment of protein sequences encoded in the mitochondrial genomes of grass species.

The amino acid sequences of mitochondrial genome-encoded proteins (1) NAD6, (2) NAD9 and (3) RPS4 in the mitochondrial genomes of eight grass species (*H. vulgare*, *T. timopheevii*, *Ae.* speltoides, *L. perenne*, *S. bicolor*, *Z. mays*, *O. sativa* ssp*. japonica* and *O. sativa* ssp. *indica*) were aligned.

Figure S1

(1) NAD6


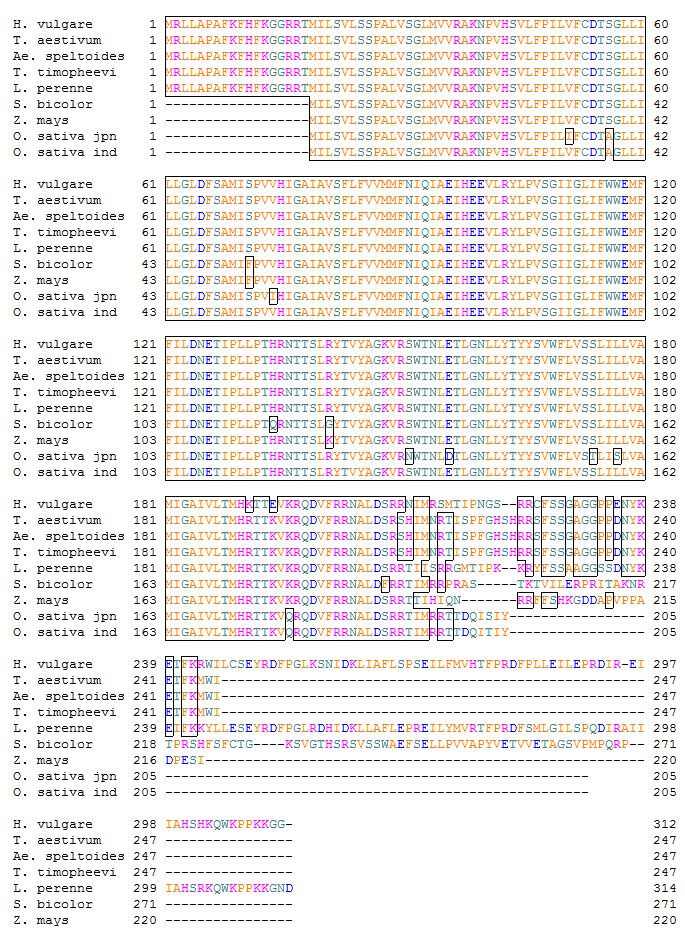


(2) NAD9


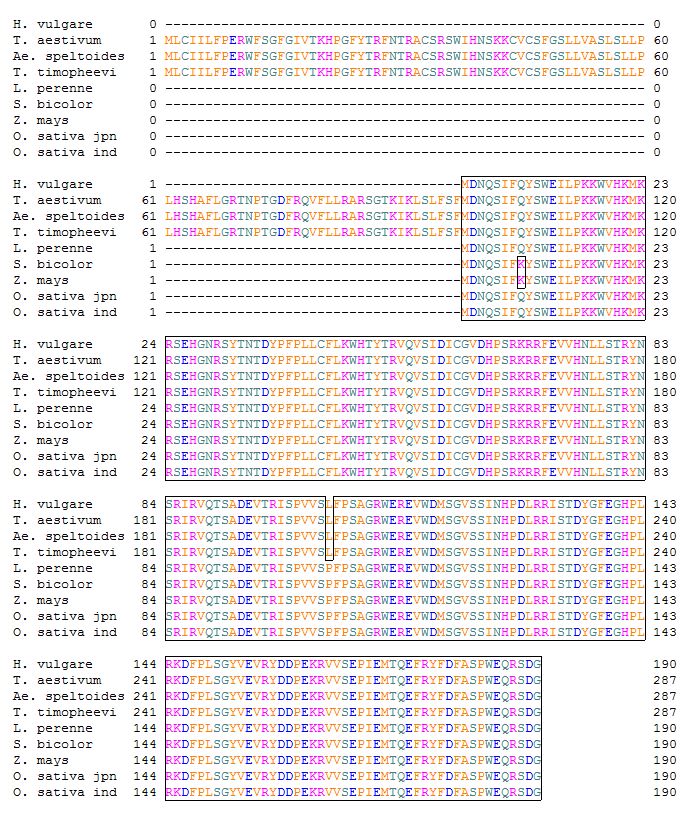


(3) RPS4


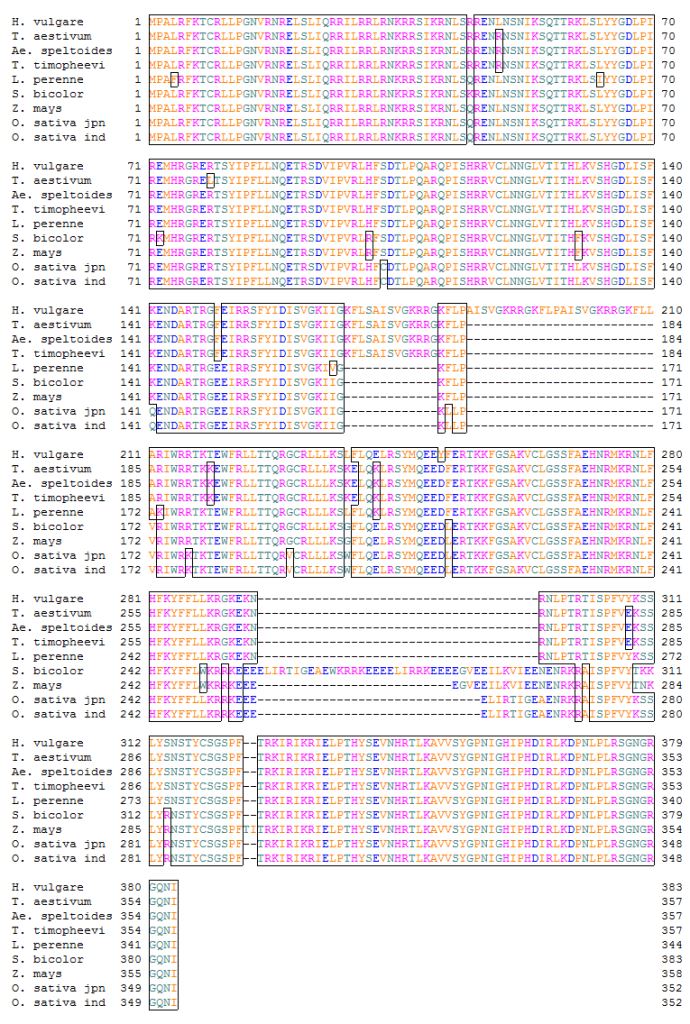

Supplement: Additional file 10: Figure S1. — Alignment of protein sequences encoded in the mitochondrial genomes of grass species. (DOC 740 kb) [file 12864_2016_3159_MOESM10_ESM.doc]
